# The Canine Erythrocyte Sedimentation Rate (ESR): Evaluation of a Point-of-Care Testing Device (MINIPET DIESSE)

**DOI:** 10.1155/2020/3146845

**Published:** 2020-08-06

**Authors:** Carlo Militello, Anna Pasquini, Anyela Andrea Medina Valentin, Petra Simčič, Giulia De Feo, George Lubas

**Affiliations:** Department of Veterinary Science, University of Pisa, Pisa, Italy

## Abstract

The erythrocyte sedimentation rate (ESR) in canine medicine has been replaced by the evaluation of a few sensitive markers of the acute-phase proteins. The aim of the study was to evaluate the ESR using a point-of-care (MINIPET, DIESSE Diagnostica Senese S.p.A.) device (ESR-MP) and to compare the results with the gold standard Westergren method (ESR-W) in dogs. One hundred and nineteen K3-EDTA blood samples for complete blood count were randomly selected and assayed for ESR. The reference interval (RI) was established using the percentile method. The coefficient of variation (CV) in intra-assay and interassay precision of ESR-MP was calculated. The analytical sensitivity (Se), specificity (Sp), positive predictive values (PPVs), and negative predictive values (NPVs) were calculated. Agreement between ESR-MP and ESR-W was assessed with Pearson correlation coefficient (*r*), Cohen concordance test (*K*), Passing-Bablok regression, and Bland–Altman plots. Ten canine samples (8.4%) were ruled out because of flag-error by the MINIPET instrument (4.2%) or because they showed the diphasic pattern in ESR-W (4.2%). The canine RI of ESR-MP was 0–10 mm/h. Precision was excellent in intra-assay (CV = 0.02) and interassay (CV = 0.32). The analytical characteristics of ESR-MP in nonanemic samples were as follows: Se = 0.82, Sp = 0.95, PPV = 0.82, and NPV = 0.95. The accuracy of ESR-MP was better than ESR-W in nonanemic samples (*r* = 0.87; *K* = 0.77) and lower in anemic subjects (Hct <37%) (*r* = 0.76; *K* = 0.69). The Passing-Bablok regression showed the presence of systematic error and the absence of proportional error only in nonanemic blood samples. The Bland–Altman plots gave negative average values due to the difference in RIs and an agreement in both ESRs. The ESR-MP results can be obtained with the same K3-EDTA tubes used for the blood count, in shortcut time, and at reduced costs using the MINIPET device. These investigations highlight that ESR-MP could be useful in canine clinical settings.

## 1. Introduction

The erythrocyte sedimentation rate (ESR) is one of the most widely performed laboratory assays in human medicine because it highlights the occurrence and the extent of inflammation. It is based on the principle that the sedimentation of red blood cells in autologous plasma is faster in patients with an increased plasma concentration of certain proteins, generally associated with acute tissue damage, chronic inflammation or infection, malignancy, and pregnancy [1]. In fact, the rates of aggregation and sedimentation are manifestations of the blood suspension instability, based on a reciprocal effect between the erythrocyte membrane surface and plasma proteins called “agglomerans” such as fibrinogen, immunoglobulin M (IgM), and alpha-2-macroglobulin [2]. Other factors influencing the ESR are primarily linked to the hematocrit (Hct) value and, in general, to blood interferents such as lipemia and hemolysis [2].

In human medicine, the ESR is commonly used as a generic sickness index in conjunction with the patient's clinical history, physical examination findings, and clinicopathological results. In addition, its clinical utility has been demonstrated in human medicine in primary care assistance, geriatric patients, hospitalized patients, hematological malignancies, stroke, heart disease, as well as in several inflammatory conditions of the osteomuscular system, and also cancer [3].

The procedure was initially described in 1894 by Edmund Biernacki, as well as independently thereafter by Drs. Hirszfeld, Fåhraeus, and Westergren [4]. The original Westergren method is the gold standard to perform ESR measurement and most laboratories (about 72%) currently adopted various modified versions because they are often significantly faster, safer, and less labour intensive [5].

In veterinary medicine, ESRs were once used but the clinical evidence of inflammation is currently based on the evaluation of some specific and sensitive markers included in acute phase proteins (i.e., C-reactive protein, haptoglobin, and fibrinogen) [6–8].

The aim of this study was to evaluate the ESR values in dogs obtained from a modified Westergren method by MINIPET in comparison with the gold standard original Westergren method and to establish the reference interval.

## 2. Materials and Methods

### 2.1. Blood Samples, Data Collected, and Equipment

This prospective study included canine blood samples, collected in 1 mL K3-EDTA tubes (APTACA Spa, Canelli, AT, Italy), the diameter and length were 12 mm and 56 mm, respectively, used primarily for blood counts, from patients that were selected randomly from the population referred to the Veterinary Teaching Hospital from February 2017 to March 2018. An informed consensus statement was signed from each dog owner to use the samples for this study. Each sample was assayed using MINIPET (ESR-MP) and the gold standard Westergren method (ESR-W) within four hours of blood collection. Any blood sample showing any apparent degree of lipemia or hemolysis was discarded and not used in the experiment.

To perform the ESR-MP, a MINIPET device (DIESSE, Diagnostica Senese S.p.A., Siena, Italy) was used. The MINIPET is an automatic continuous loading instrument analysing up to four blood samples simultaneously collected in standard K3-EDTA tubes, using an optical system that measures the erythrocytes sedimentation level. The data are then processed and printed or appear on a display. This method enables the use of the same sample tubes used for the blood count (K2-EDTA or K3-EDTA vials with the size as above of different brands) and also provides results (reported in mm/h), corrected at the temperature of 18°C according to Manley's nomogram, in 20 minutes [9].

To perform the original Westergren method (ESR-W), the Takives pipettes with the appropriate stand (Biosigma, Cona, VE, Italy) were used [3].

For each blood sample tested, the Hct value was assessed by a ProCyte Dx® hematology analyzer (IDEXX Laboratories Inc., Milan, Italy).

All the blood samples collected were divided into three groups to evaluate the interference of anemia in the blood samples: group 1, all blood samples; group 2, nonanemic blood samples (Hct ≥ 37%, range: 37.0–57.6%); and group 3, anemic blood samples (Hct < 37%, range: 10.2–36.7%).

### 2.2. Statistical Analyses

All data, except for those showing the message “ERR” (Error) from ESR-MP or with diphasic pattern in ESR-W, were analysed [10]. The reference interval (RI) for ESR-MP was determined, using data from blood samples of group 2 (HCT ≥ 37%) showing a physiological ESR-W value (≤5 mm/h) [11, 12]. These data were tested for normality distribution using the Shapiro–Wilk test, and they resulted to be non-normally distribuited, and the RI was assessed using the percentile method (2.5^th^–97.5^th^). The intra-assay precision or within-run repeatability (the minimum of three blood samples measured eight times in each session) and the interassay precision or between-run reproducibility of ESR-MP (double reading of 80 blood samples in about 20 working days) were performed, and the coefficient of variation (CV) were calculated [13]. The analytical agreement for ESR-MP and ESR-W was assessed by sensitivity (Se), specificity (Sp), negative predictive values (NPVs), and positive predictive values (PPVs). The agreement between ESR-MP and ESR-W was assessed using the Pearson correlation coefficient (*r*). Cohen's Kappa test was used in order to evaluate the strength of agreement to categorize correctly an animal with an abnormally high or physiological value between the results obtained by ESR-MP and those by ESR-W. Finally, the statistical methods recommended for the validation of alternative ESR methods by the International Council for Standardization in Hematology (ICSH) such as the Passing-Bablok regression and the Bland–Altman plots between ESR-MP and ESR-W were used, after consulting few other papers [3, 5, 14]. All data were analysed using Microsoft Excel® 2016 Software, MedCalc® Portable Software 2015, and the R Project for Statistical Computing.

## 3. Results

A total of 119 blood samples were collected. Ten samples (8.4%) were ruled out because of an error (ERR) flag by the MINIPET (4.2%) (Hct values ranging 10.2–36.7%), or due to a diphasic pattern in ESR-W (4.2%) (Hct values ranging 15.4–44.7%).

A total of 57 blood samples from among the 76 samples of group 2 matched the inclusion criteria and were suitable for measuring the ESR-MP reference interval. The reference interval of ESR-MP was established as 0–10 mm/h. The intra-assay and interassay coefficients of variations were 0.02 and 0.32, respectively.

**Figure 1:**
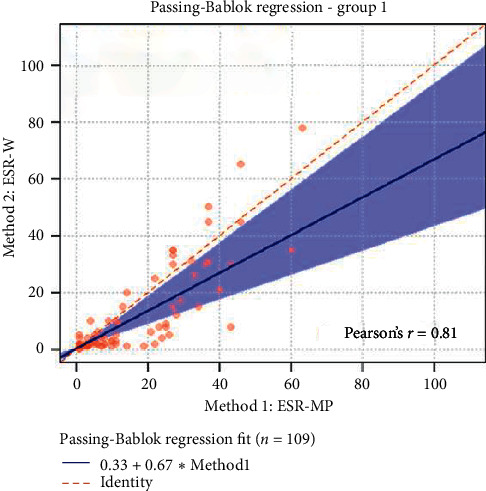
The Passing-Bablok regression of group 1 (all blood samples collected). ESR-W, erythrocyte sedimentation rate using the Westergren method; ESR-MP, erythrocyte sedimentation rate using the MINIPET device. Additional information: intercept-systematic difference was 0.33 (95% CI: 0.31 to 0.57); slope-proportional difference was 0.67 (95% CI: 0.42 to 0.98).

**Figure 2:**
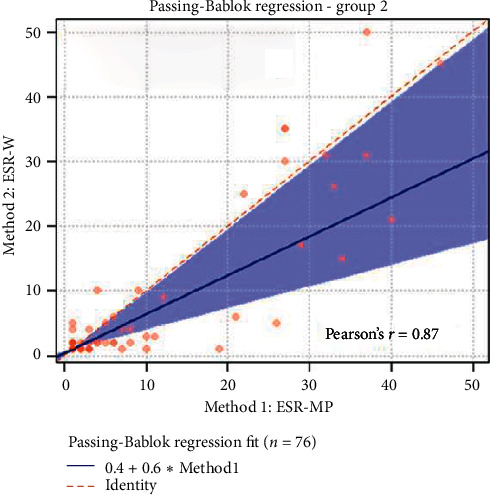
The Passing-Bablok regression of group 2 (nonanemic blood samples). ESR-W, erythrocyte sedimentation rate using the Westergren method; ESR-MP, erythrocyte sedimentation rate using the MINIPET device. Additional information: intercept-systematic difference was 0.40 (95% CI: 0.02 to 0.67); slope-proportional difference was 0.60 (95% CI: 0.33 to 0.98).

**Figure 3:**
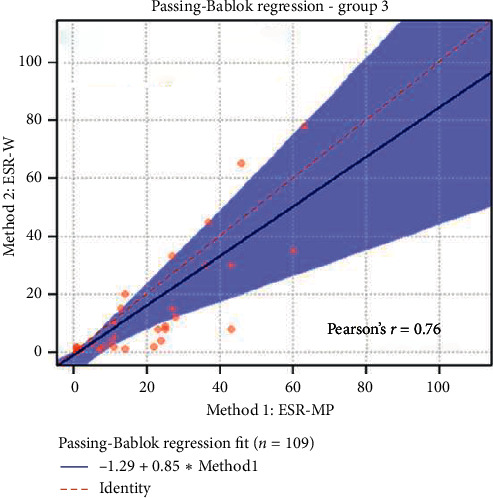
The Passing-Bablok regression of group 3 (anemic blood samples). ESR-W, erythrocyte sedimentation rate using the Westergren method; ESR-MP, erythrocyte sedimentation rate using the MINIPET device. Additional information: intercept-systematic difference was 1.29 (95% CI: 9.43 to −0.16); slope-proportional difference was 0.86 (95% CI: 0.44 to 1.38).

**Figure 4:**
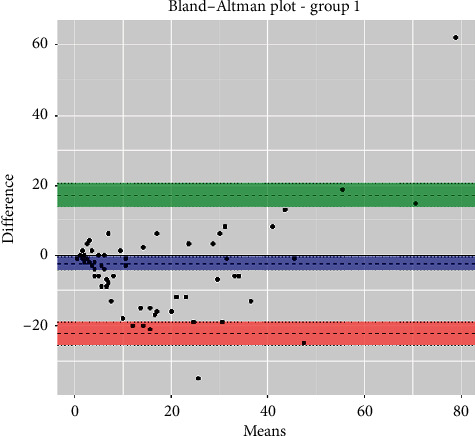
The Bland–Altman plot of group 1 (all blood samples collected). Additional information: upper limit was 17.4 (95% CI: 14.0 to 20.7) (green); mean difference was 2.4 (95% CI: 4.4 to −0.5) (blue); lower limit was 22.3 (95% CI: 25.6 to −19.0) (red).

**Figure 5:**
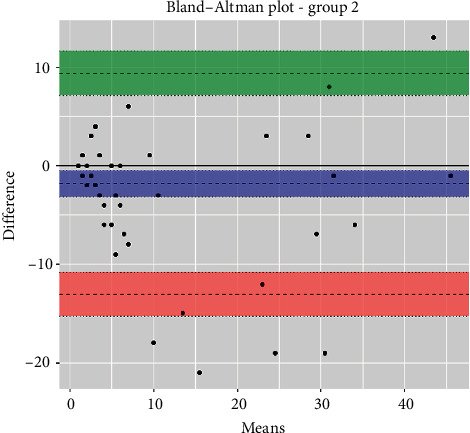
The Bland–Altman plot of group 2 (nonanemic blood samples). Additional information: upper limit was 9.4 (95% CI: 7.1 to 11.6) (green); mean difference was 1.8 (95% CI: 3.2 to −0.5) (blue); lower limit was 13.0 (95% CI: 15.3 to −10.8) (red).

**Figure 6:**
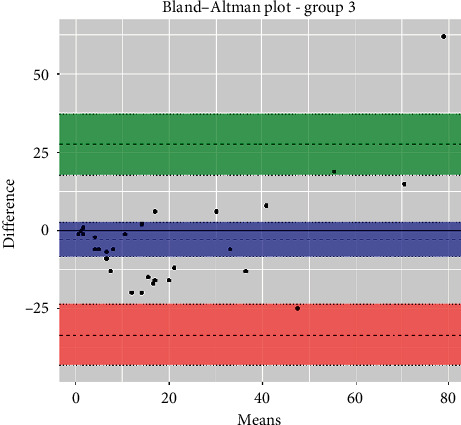
The Bland–Altman plot of group 3 (anemic blood samples). Additional information: upper limit was 28.1 (95% CI: 18.0 to 38.1) (green); mean difference was 3.9 (95% CI: 9.7 to 1.9) (blue); lower limit was 35.9 (95% CI: 45.9 to 25.9) (red).

The statistical analyses, i.e., analytical sensitivity, specificity, PPV, and NPV, are reported in Table 1 along with the Pearson correlation coefficient and Cohen's Kappa test. The Passing-Bablok analysis for the three groups is reported in Figures 1–3. The Bland–Altman plots for the three groups are reported in Figures 4–6.

## 4. Discussion

The ESR is generally higher in acute general and localized inflammation and is used as a nonspecific screening test to detect the acute phase of the inflammatory response and to monitor chronic diseases. The ESR provides evidence of pathological conditions and depends on a large number of variables [1].

The ESR can be measured with two main methods: the Westergren and the Wintrobe. The latter was used at the beginning in the 1970s in veterinary medicine [6, 7]. Later on, the Westergren method became the gold standard in human medicine thanks to ICSH recommendations, and it was also adopted in veterinary medicine [11]. Even though, in human medicine the, ESR remains the most widely performed laboratory assay, there is a lack of ESR studies in veterinary medicine compared to human medicine.

Today, the Westergren method still remains the benchmark in human medicine [2]. Only 28% of laboratories surveyed used the unmodified Westergren method, while 72% of sites used modified or alternate methods which are often significantly faster, safer, and less labour intensive [5]. A new automatic instrument, such as the ESR-MINIPET, could encourage the use of ESR in canine clinical veterinary medicine. In addition, the ESR-MINIPET is rapid, providing the result in 20 min, and safe since it uses the same blood tubes as a full blood count test, without the risk of aerosol and of any additional sampling.

In our study, all the blood samples showing an error message from ESR-MP had a high value on ESR-W. Moreover, the diphasic pattern found in our study occurs occasionally where there is no clear line or separation between the settling of erythrocytes and the plasma observed in the sample tube. The pattern is, in fact, due to the presence of reticulocytes or other younger RBCs besides nucleated erythrocytes. In addition, a diphasic pattern could occur if there are a great number of abnormally shaped erythrocytes, in which case a stained blood smear should be examined at the microscope and the reticulocyte count should be assessed [11].

This study was prepared according to the guidelines presented in a recent paper on the quality assurance and standards in Veterinary Clinical Pathology regarding the method of validation and verification of this modified method as well as the ESR-MP [13]. In addition, the paper from Jensen and Kjelgaard-Hansen about the method comparison in the clinical laboratory specifically directed to veterinary clinicopathological data was also consulted [14].

The RI established using the ESR-MP was different from the ESR-W and it was twice the range (0–10 mm/h vs. 0–5 mm/h). The results obtained in intra- (0.02) and interassay (0.32) coefficients of variations ensure that the possibility can be repeated accurately. The analytical sensitivity, specificity, negative predictive values, and positive predictive values were higher than 0.80 in all subgroups, except in anemic blood samples where lower values both in the sensitivity and negative predictive values were obtained. In addition, a value of 1.00 in both specificity and positive predictive values in anemic blood samples was observed.

The highest correlation value between the ESR-MP and ESR-W was obtained in nonanemic blood samples (*r* = 0.87), while the lowest value was obtained in anemic blood samples (*r* = 0.76). The strength of agreement of the data was assessed by Cohen's Kappa test and was good in all three subgroups according to Altman, 1991 (0.61–0.80), giving another view of the use of ESR-MP in clinical settings [15]. Note that the ESR results are influenced greatly by the Hct values, indeed specific nomogram to correct the ESR value based on Hct and/or Hgb value both in humans and dogs has been proposed [10, 16, 17]. In this paper, the corrected ESR value based on Hct was not considered, instead the original results were analysed and compared.

In the Passing-Bablok regression, systematic errors were present in both nonanemic and anemic blood samples, and there were no proportional errors only in anemic blood samples. In the Bland–Altman plots, a negative average value was found in all the subgroups, which reflected a wider interval reference compared to ESR-W, and there was an agreement between the two ESR methods since almost all the points were within the reference interval.

The study presented some limitations that should be investigated further such as the influence in the sample submitted to ESR-MP about the common preanalytical errors (i.e., hemolysis, icterus, and lipemia), which were not investigated, the diphasic ESR, and the Hct values. Indeed, in anemic samples, the ESR should be corrected in relationship to the Hct values and an appropriate formula should be arranged.

## 5. Conclusions

The MINIPET method is as reliable and efficient as the ESR-W in identifying a high or physiological value of the ESR in nonanemic samples. It also confirms the presence of high ESR in anemic blood samples, which should be interpreted based on the Hct values. The established reference interval for ESR-MP is 0–10 mm/h, thus reflecting the physiological values.

We believe that in order to establish a correct nomogram to best interpret the ESR, the reference interval of MINIPET represents a starting point to investigate ESR-MC in samples collected from diseased animals including those with Hct below the reference interval. It would also be interesting to investigate whether the ESR can be used to diagnose the occurrence of infectious, immune-mediated, and neoplastic diseases in canine medicine as effectively as in human medicine [18]. Additionally, it would be useful to evaluate ESR-MP in terms of its prognostic value and effectiveness in monitoring the course of several disorders [19–21]. The new safe, automatic, and fast instrument, ESR-MINIPET, could help to promote ESR once again in the clinical veterinary medicine.

## Figures and Tables

**Table 1:** Statistical analyses of two ESR methods (ESR-MP and ESR-W) compared each other for the three groups of blood samples.

	Group 1	Group 2	Group 3
Number of samples	109	76	33
Sensitivity	0.80	0.82	0.77
Specificity	0.96	0.95	1.00
PPV	0.91	0.82	1.00
NPV	0.89	0.95	0.69
*r*	0.81	0.87	0.76
*K*	0.77	0.77	0.69

Note: group 1, all blood samples collected; group 2, nonanemic blood samples; group 3, anemic blood samples; PPV, positive predictive values; NPV, negative predictive values; *r*, Pearson correlation coefficient; *K*, Cohen's Kappa test.

## Data Availability

The data supporting the conclusion of the study can be made available on request to the corresponding author as the device MINIPET DIESSE is patented.
